# A Curious Case of Autoimmune Pancreatitis with IgG4-related Sclerosing Cholangitis

**DOI:** 10.7759/cureus.4153

**Published:** 2019-02-28

**Authors:** Hector H Gonzalez, Jamie L Skrove, Seth Rosen, Javier Sobrado

**Affiliations:** 1 Internal Medicine, Florida Atlantic University Charles E. Schmidt College of Medicine, Boca Raton, USA; 2 Internal Medicine, Larkin Community Hospital, South Miami, USA; 3 Gastroenterology, Larkin Community Hospital, South Miami, USA

**Keywords:** autoimmune pancreatitis, igg4-related disease

## Abstract

Immunoglobulin G4-related sclerosing cholangitis (IgG4-SC) is a novel entity that belongs to the immune-mediated fibroinflammatory class of IgG4-related diseases (IgG4-RD). IgG4-SC is noted to be one of the most frequent manifestations of extra-pancreatic disease among IgG4-RD, which is significantly different from primary SC (PSC) and cholangiocarcinoma (CC) as is evident in the varied approaches to treatment. IgG4-RD includes IgG4-SC and autoimmune pancreatitis (AIP).

Herein, we presented a case of IgG4-SC in a patient with obstructive jaundice secondary to AIP. We have also discussed the current recommendations for diagnostic and treatment modalities, with an emphasis on the issues that arise in obtaining a definitive classification of disease.

## Introduction

IgG4-related sclerosing cholangitis (IgG4-SC) is an uncommon condition recently recognized as an independent disease entity in hope to better specify future diagnosis modalities and treatment. Its exact pathophysiologic mechanism is yet to be identified, but evidence of submucosal fibrosis and inflammatory cells infiltrating biliary ducts has been noted on biopsies [1-2]. Herein, we presented a case of IgG4-SC in a patient with obstructive jaundice secondary to autoimmune pancreatitis (AIP).

## Case presentation

**Figure 1:**
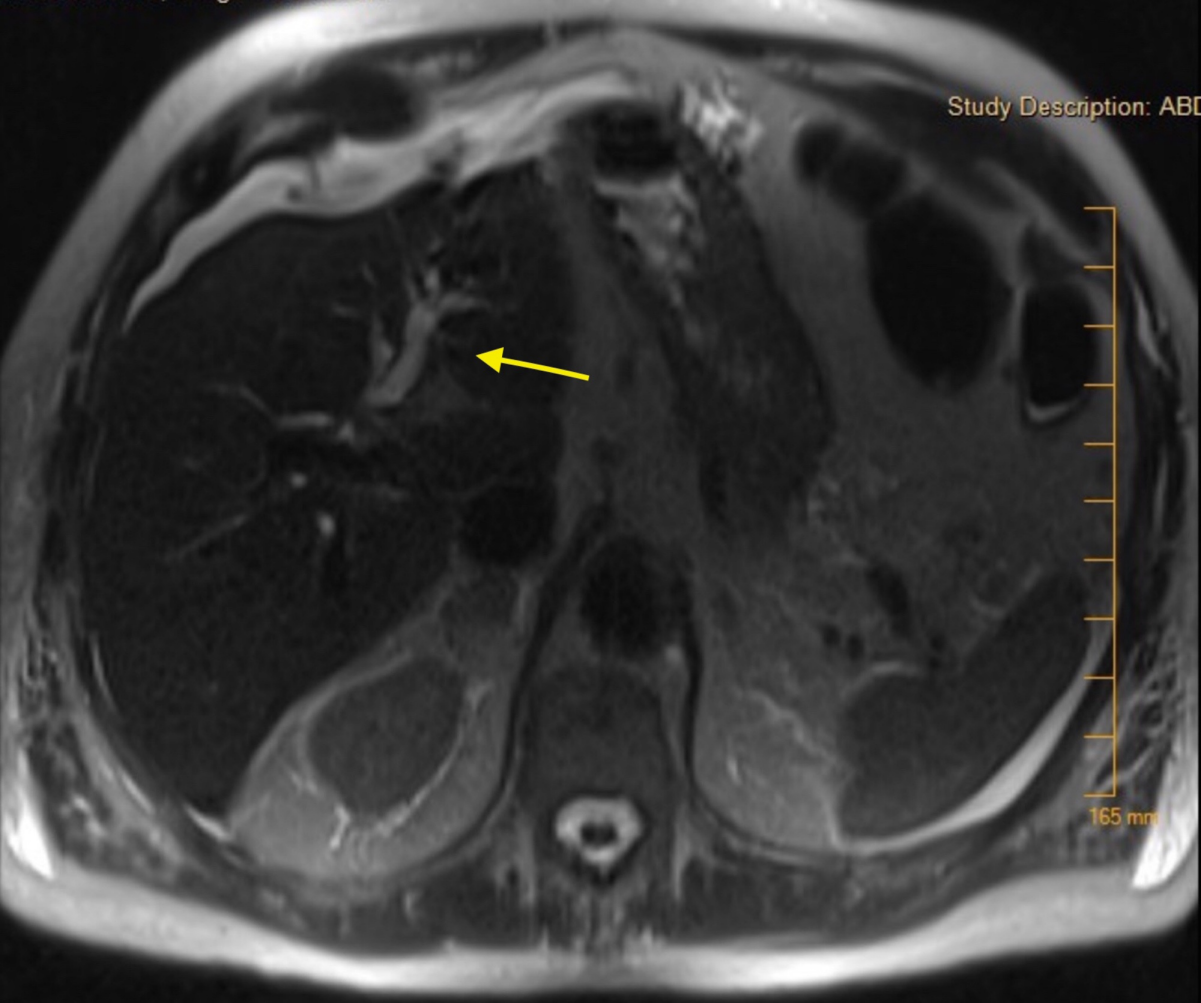
MRI of the abdomen and pelvis demonstrating dilation of biliary ducts (arrow) MRI: magnetic resonance imaging

**Figure 2:**
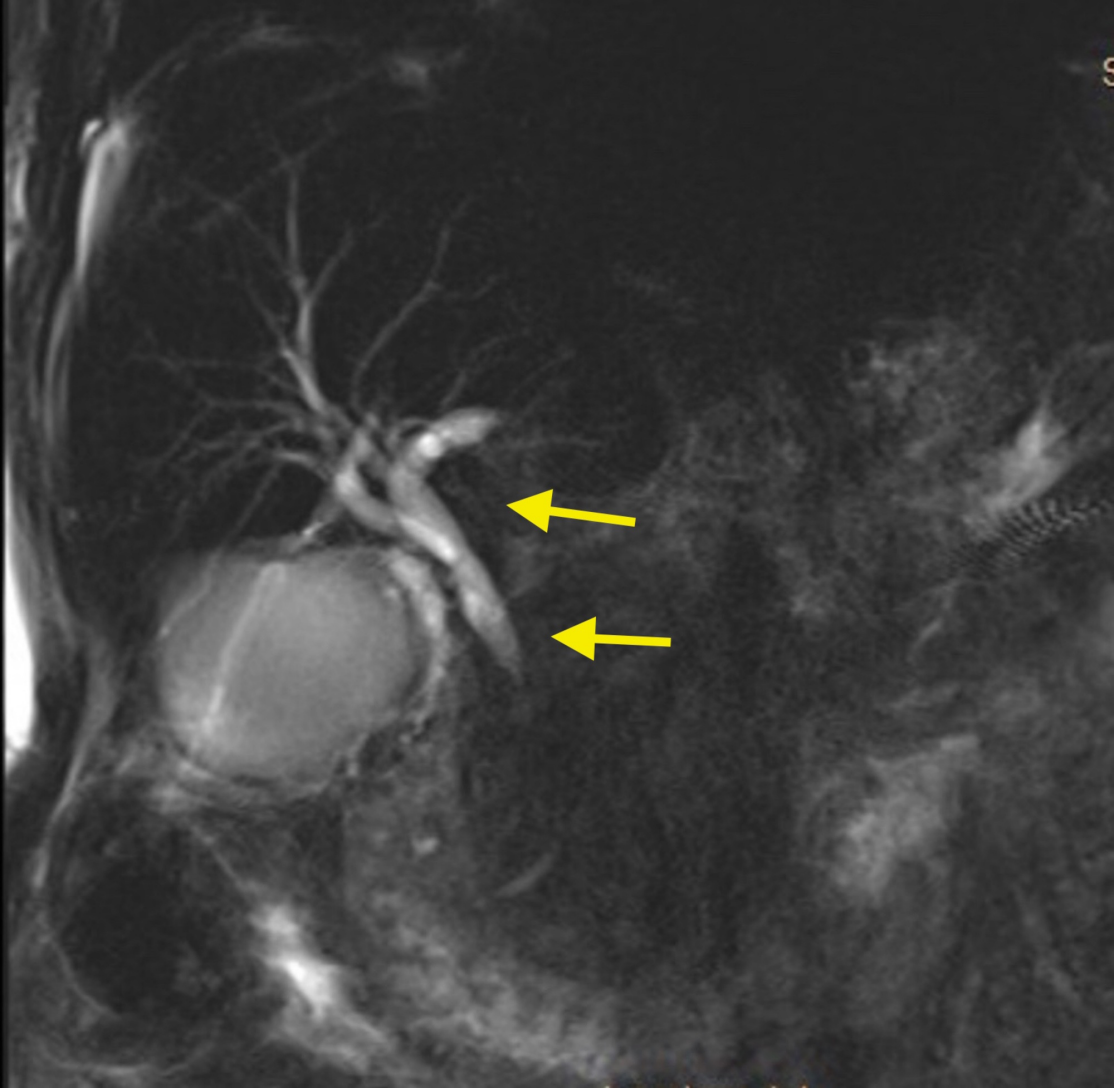
MRI of the abdomen and pelvis showing intra-hepatic bile duct dilation with common bile duct narrowing (arrows) MRI: magnetic resonance imaging

The patient is a 54-year-old male with no known past medical history who presented to the hospital with sudden onset of sharp, epigastric abdominal pain, weight loss, and nausea. Physical examination was remarkable for epigastric tenderness, scleral icterus, and painless jaundice. He was admitted to the hospital after being stabilized with intravenous saline, antiemetic medication, and analgesics. Liver function tests were elevated, Carcinoembryonic antigen (CEA) level was 652.9 ng/ml, and IgG4 was 464mg/dL. Computed tomography (CT) scan of the abdomen and pelvis revealed diffuse parenchymal enlargement, with surrounding inflammatory changes. Magnetic resonance imaging (MRI) of the abdomen revealed heterogeneous enhancement in the head of the pancreas along with a short segmental stricture of the common bile duct with extrahepatic biliary dilatation (Figures 1-2). 

Liver biopsy was performed with hematoxylin and eosin stain showing cuff-like periductal lymphoplasmacytic infiltration and normal surrounding pancreatic parenchyma. Plasma cell-rich mixed infiltrate around bile ducts and periductal fibrosis were noted as well. These biopsy findings along with serum IgG4 levels were consistent with IgG4 AIP. The patient responded well to steroids, and the CEA levels dropped. He followed up in office and steroids were tapered with plans to start azathioprine. Ten months later, he presented to the hospital with obstructive jaundice and right upper quadrant pain. IgG4 at that time was >700 ng/ml. MRI of the abdomen and pelvis showed relapse of AIP with cystic changes at the level of the pancreatic neck, as well as a 1-cm long stricture of the proximal intrapancreatic portion of the common bile duct, wall thickening of the common hepatic duct, and the common bile duct. These findings were indicative of sclerosing cholangitis or IgG4-SC. The patient was again started on steroids and is currently doing well.

## Discussion

Immunoglobulin G4-related diseases (IgG4-RD) consist of a spectrum of diseases including IgG4-SC and AIP [1-2]. The classic clinical presentation among patients is multi-organ involvement of disease with a prevalence noted in middle to older-aged males [1-4]. Clinicians should maintain significant suspicion especially in the setting of extra-biliary manifestations such as parotid/lacrimal swelling, lymphadenopathy, AIP, and retroperitoneal fibrosis [5-6]. The presence of a certain constellation of features such as elevated IgG4 levels, abnormal imaging, and biopsy findings (IgG4+ plasma cells, lymphoplasmacytic infiltrate, storiform fibrosis, or obliterative phlebitis) suggest disease [6-9]. To date, IgG4-RD seemed to respond favorably to systemic glucocorticoid therapy.

AIP is one of the most common extra-biliary etiologies of IgG4-RD. There are two distinct subtypes classified as type I and type 2 AIP [10-11]. Type I AIP is also referred to as IgG4-related pancreatitis, as opposed to type II that is unrelated to underlying IgG4-RD [9]. The typical clinical presentation for these patients includes intractable nausea/vomiting, generalized abdominal pain, palpable abdominal mass, jaundice, recurrent pancreatitis, and alteration in weight [4-5,7-9,11]. The literature suggests a predisposition to the development of AIP in patients with concomitant autoimmune diseases such as Hashimoto disease [7,11]. When considering the diagnosis of AIP, it is important for clinicians to maintain a differential for possible underlying pancreatic malignancy. The presence of intrapancreatic stenosis should prompt further diagnostic studies to exclude cholangiocarcinoma (CC) or pancreatic malignancy. The diagnosis of AIP can be made by five criteria of histology, imaging, serology, other organ involvement, and response to steroid therapy defined by the HISORt criteria [4-5,7-9,11]. Our patient was initially diagnosed and treated for AIP, with the development of IgG4-SC as late sequelae. These two conditions are not mutually exclusive; in fact, much of the literature explores a possible underlying association. There are no trials conducted that have made a consensus regarding appropriate treatment for patients with AIP. However, there are reports of AIP responding favorably to a short course of glucocorticoid therapy. Cases that are refractory to an initial short course of steroids have shown to respond well with repeat short course or low maintenance dose therapy [7-11].

IgG4-SC is a novel entity that has been documented to be associated with conditions such as autoimmune pancreatitis, primary SC (PSC), and CC [1-2]. There is an increased prevalence in men, the elderly, and patients with multiorgan involvement [1-2,4-6]. Several factors have been used for the diagnosis such as IgG4 level, cholangiography, and IgG4-positive plasma cell infiltration on biopsy [1-2,9-10]. When performing cholangiographic evaluation of patients suspected of having IgG4-SC, clinicians must remain cognizant of alternative diagnoses, particularly PSC and CC. The presence of inflammatory bowel disease in the setting of imaging findings of biliary disease would suggest a possible underlying component of PSC. With the considerable biliary disease burden seen with PSC, often times, the diagnosis of IgG4-SC may go overlooked. Furthermore, clinicians should maintain suspicion for possible CC in the presence of segmental stenotic regions on cholangiography. Despite the fact that little is known regarding treatment options, IgG4-SC has been documented to respond to steroids (Poster: Gonzalez H, Skrove J, Colella D, Rosen M, Sobrado J. A Curious Case of Autoimmune Pancreatitis with IgG4 Related Sclerosing Cholangitis. ACG annual meeting; 2018). The precise dose of steroids to be utilized is yet to be established. Alternative treatment modalities consist of biliary stent placement, drainage, or surgical intervention [1,4-5].

## Conclusions

IgG4-SC is a novel entity that has been documented to be associated with conditions such as AIP, PSC, and CC. Due to the overlapping clinical presentation of IgG4-SC with other disease processes, prompt diagnosis, classification, and appropriate treatment modalities are difficult to establish. To date, several diagnostic criteria have been proposed as a means to help identify IgG4-SC and other related diseases. However, further comparative diagnostic and treatment studies are needed with treatment remaining inconsistent and a lack of formally established diagnostic criteria.
